# Ten Months in France with a Field-Service X-Ray Outfit

**Published:** 1916-12

**Authors:** William Cotton

**Affiliations:** In charge X-ray Department, No. — General Hospital B.E.F.


### TEN MONTHS IN FRANCE WITH A FIELD-SERVICE X-RAY OUTFIT.

BY

William Cotton, Capt., R.A.M.C. (T.F.),

*In charge X-ray Department, No. — General Hospital B.E.F.*

Having spent more than eight months of the war in Bristol waiting for work with the sick and wounded, I finally got the permission of my local Commanding Officer, and applied direct to the War Office, offering to go in charge of any X-ray department in any military hospital at home or abroad, and to make myself generally useful. In less than a week I received a telegram ordering me to mobilise next day at a rest camp with a general hospital then forming. I had relied on some practical knowledge of X-ray photography in the very early days of the art, and also on the concoction, from time to time, of over twenty-five papers on the interpretation of X-ray pictures. I found in the sequel that my somewhat theoretical investigations were most of them highly intelligent anticipations of the best every-day practice of the present time, though others of them still await practical realisation, more especially in the application of proportional representation to X-ray medical diagnosis.

My arrangements had long been made for any sudden summons, and I promptly reached the rendezvous, where I had an interview with my new Commanding Officer, who gave me a wigging for being in mufti, an identity disc "to secure a Christian burial," and two days' leave back to Bristol to obtain my uniform and kit.

On my return I was billeted at a comfortable hotel in the town, and spent my time in making acquaintance with my new comrades, in airing my new clothes, in drilling (and grilling) on the gorse-surrounded glades of the common, in picking up the routine of duty, in receiving lectures from one of our regular majors on the science and art of filling up diet sheets, and in strolling about the charming environs of a very pretty seaside town. Also I had myself to lecture on the sward to different squads of our rank and file. Luckily I had with me a few lantern slides and carbon reductions of X-ray photographs, which rendered my prelections, I hope, more objectively interesting. From individual listeners I had many useful suggestions and reminiscences. Blessed by delightful weather and with all our beautiful surroundings our last days in England passed pleasantly and swiftly by.

Life in the army is in the present war much of a lottery, by the trick of a number we might have gone farther and fared worse. As it was, our turn came to march in at the dock gates and to embark on the waiting transport. The next morning, after an uneventful passage, we found ourselves anchored off a foreign seaport, and a little later tied up to a quayside that must have been familiar to many of us. The first night we spent on ship-board with the cargo cranes banging all night, and the second at a rest-camp on a hill, where I had my first experience of sleeping under canvas, and very cold at that. On our way to entrain thence we passed below some ramparts, where the visitor is shown the bench on which Pendennis the younger found his dear old colonel sitting, when he went to succour him in his foreign refuge. Finally, a short and slow railway journey landed us all at our destination, a roadside station between sandy dunes and verdant chalky downs, upon a partially cultivated green veldt at the side of the railway line. A few bell tents without floors, one or two marquees, and a skeleton hut here and there were all there was then to mark the site of what in a few weeks was a canvas town of hospital tents and offices to hold thousands of patients, hundreds of attendants, and scores of physicians and surgeons.

Arriving on the ground on May 16th, 1915, our hospital, including the X-ray department, was ready for its full complement of patients by June 4th. Our first X-ray home was a field operation marquee, with a very limited and uneven floor-space, much encroached on by its slanting sides, with just room, in fact, for an X-ray table and our coil and accumulators on a wheeled trolly. We had changed our brand-new uncharged accumulators for an ancient charged set at the Base Medical Stores, and took our first X-ray photograph on June 5th. In about a week we removed to the X-ray compartment of the general operating hut next to the theatre, practically an open-air shed, until we removed to our present really commodious and comfortable X-ray hut attached to the officers' surgical ward. In spite of many technical and climatic difficulties (exposed as we were to heat, cold, damp, rain, dust, glare, and absence of artificial light and warmth, till some seven months had elapsed), I am glad to say we never had to refuse any case brought to us for any but an hour or two's temporary breakdown.

The work in the ten months under review was done entirely by accumulators. In that time we photographed 1,607 patients on 2,849 plates, taking 1,954 different parts, all cases of British soldiers, with the exception of a few nurses and 14 French local patients.

On 47 of our 304 working days we had no cases to take; these days were spent in experimenting on our connections and tubes, overhauling our apparatus generally, and in taking down and moving and re-erecting our apparatus as occasion required. On one of these off days we X-rayed stereoscopically a skull lent by the dentists, with a perfect set of teeth, antero-posteriorly from the sides and from the base, and found these photos when viewed singly most excellent key-charts in determining the landmarks of the clinically X-rayed skull. I cannot help thinking how valuable these X-ray surveys of the dried human skeleton would be generally, especially of such joints as the shoulder and hip, from different points of view. The average daily number of plates taken was over 9; one day we took 75, often 40 and 30, and very often 20. So far as the statistics of our unit are available, we examined by X-rays two-fifths of the surgical cases admitted.

Probably the accompanying tables will give in brief the best idea of the amount of work, and some idea of its nature, requiring to be done at a base hospital at the back of the front on the lines of communication in Northern France. The vast majority of cases admitted were recent gun-shot wounds, many infected at the time of injury. Most of the fractures were compound and comminuted. It is evident from the present tables that many patients had more than one injury, affecting more than one part, *e.g.* one had to be photographed on six different plates, over seven different parts. Having a special dental department, we had many cases of injury to jaws and face. Many cases were X-rayed at intervals to test progress and results :—

### TABLES.

### Patients Examined.

| | |
|---|---|
| British .. .. .. .. .. | 1,593 |
| Local French .. .. .. .. | 14 |
| Total .. .. | 1,607 |

### Plates Taken.

| | |
|---|---|
| (*a*) Not sufficiently clear to give certain assurance of presence or absence of a lesion ; that is, poor plates from insufficient penetration .. .. | 103 |
| (*b*) Sufficiently clear to negative presence of an X-rayable lesion .. .. | 826 |
| (*c*) Giving positive evidence of presence of one or more definite lesions .. | 1,920 |
| Total .. .. | 2,849 |

(including single " view finders, 997 ; 76 stereoscopic pairs ; 370 pairs of plates of the same part taken at planes at right angles to each other, and 31 exact mathematical localisations by double exposure on one plate).

PARTS TAKEN (Class (*c*) only).

| | | | |
|---|---|---|---|
| Hands and Wrists | 145 | Thighs | 107 |
| Forearms | 113 | Hips, ilium and ischium | 50 |
| Elbows | 82 | Neck | 27 |
| Upper Arms | 74 | Chest and Back | 68 |
| Shoulder, Clavicle and Scapula | 140 | Loins and Abdomen | 18 |
| Feet and Ankle | 88 | Pelvis | 22 |
| Legs | 84 | Cranium and Orbit | 110 |
| Knees | 79 | Face and Jaws | 93 |

LESIONS FOUND (Class (*c*) only).

| | |
|---|---|
| Fractures | 354 |
| Shrapnel fragments | 636 |
| Both fractures and shrapnel fragments | 86 |
| Service bullets | 44 |
| Shrapnel balls | 26 |
| Miscellaneous (including a few medical cases) | 154 |

We used all sizes of plates from $4\frac{3}{4}$ by $6\frac{1}{2}$ inches up to 12 by 15 inches; of the last in special cases we used no less than 87. They were, however, very difficult to handle and develop and wash, and except in very large patients had no advantage over our favourite size, 10 by 12 inches, which last was of great use when systematic screening was out of the question. Of every 100 plates used, 40 per cent. were either 10 by 12 inches or 8 by 10 inches. Another very useful size was 6 by 8 inches, especially with lateral views of the jaws, and in locating by double exposure on one plate, and with the hands.

The miscellaneous cases were not the least interesting of the lot, and merited more than one special monograph. At least 50 plates showed the peculiar mottling caused by the presence on the soft parts of pockets and strata containing gas and fluid infection. One skull in a case of acromegaly showed distinct enlargement of the sella turcica. Another skull showed a trephine wound. One plate showed a beautiful osteoma of the shoulder-blade. We had a case of simultaneous comminuted fracture of both fibulae, several complete dislocations of the shoulder and elbow, osteophytes about the knee-joint, a supracondyloid process of the humerus, a post-mortem specimen of a service bullet in the body of one of the upper cervical vertebræ. In one mastoid area X-rayed the shadow of a service bullet above and in front of the ear was not detected from the plate, the butt and side lines of a safety-pin obscuring the outlines of the bullet, the missile being independently found on cleaning up the wound by incision. In the stereoscopic picture of one injured shoulder the service bullet was found over the medullary canal of the humerus with the point downwards ; its position could not be determined till the stereoscopic plates were superposed, nor the surgeon satisfied till we had taken other two plates on planes at right angles to each other of the lower end of the humerus. Several patients were taken under anæsthetics, prior to ordinary operation, with various probes or forceps in the wounds to facilitate localisation. We had no undoubted case of calculus in ureter or bladder. In cases of extensive surface peppering of the soft parts with shrapnel the patients seemed to show great tolerance. I need hardly say that in the case of large surgical convoys we did a good deal of first-hand diagnosis for our surgical friends.

Our dark room work was always a bright spot ; if anything was on the plate at all, our sergeant always brought it out. As a rule, with our accumulators we got plenty of detail, but thin and unprintable negatives. It must have been no easy task to develop 40 or 50 plates on a hot summer day with water that had to be carried a quarter of a mile often. Our general duty orderly was of use through his previous knowledge of wiring and electrical connections.

Our field service outfit was of "pattern 1915," by a well-known maker. We had six lithanode accumulator batteries of three cells each. A small three-quarter horse-power engine, coupled by a chain to a small dynamo, was supplied to charge and re-charge our batteries; it was a perpetual nuisance. Too strenuous for its frame, it used to throw itself to pieces, and could be got to work only for an hour or two at a time. What happened finally I could never find out. It was rather noisy, and one fine morning I found it in its place of exile at the end of the lines motionless and alone, with a broken bed-plate and a snapped chain, in company with a smashed voltmeter and a coal hammer with a broken handle. These accumulators were exchanged for a freshly-charged set every week (latterly every fortnight) from the B. M. S.; and in the interval for every hour's discharge they got an equivalent bucking up at the camp electric lighting station, and latterly in our own hut, through a lamp resistance board. We had a wheeled trolly, for bedside work, fitted to take the accumulators underneath and the coil above. A rather better plan would have been to reverse this arrangement; as it was, the accumulators were difficult to examine and to uncover to get at a loose binding screw or the like. The use of this trolly in our hospital of irregularly distributed marquees was quite impracticable, and we used it only twice at the bedside. The coil was a 12-inch spark one and very good. We had a very good, efficient and simple control unit.

The mercury interrupter was very good, but much too complicated for accumulators, and gave much trouble and anxiety till we learnt by experience its structure and ways; with experience we got it latterly always to work very sweetly. The dialectric was condensed coal gas in cylinders; as an alternative in case of failure of supply we were instructed to use sulphuric ether, but never needed to. In some of the other hospitals hydrogen gas was made and used with success. Our table was much too elaborate, but well designed, and quite up to date. I often thought of removing the top entirely and getting the stretchers put on the frames direct.

Attached to the rolling car of the tube box was a massive upright to hold over the patient a very heavy and cumbrous overdeck, which after trial we found much too heavy for convenient habitual use. We had a very well-designed overhead tube box holder. We had an elaborately-fitted large stereoscope in book form, like a family Bible, but personally I preferred converging the vision from the front of the negatives, illuminated by daylight, against two finely-ground glass window-sashes of large size. We were plentifully supplied with all the usual accessories, namely a fluorescent screen in a bellows folding holder, changing box, intensifying screen (which we never successfully worked), iris diaphragms, short extension tubes, valve tubes, rubber gloves, and a good photographic outfit of plates and chemicals, renewed on request from the B. M. S. The four English X-ray tubes we brought with us fluoresced misty magenta, and we could get nothing out of them, and so we got rid of them by exchange, and did all our work with American tubes, which answered well. One tube I got in this way from an X-ray friend in need (and in deed) at a neighbouring hospital; it has taken over 1,500 photographs, and works as well as ever, both with accumulators and with an electric light current. Judging from my experiences here with the rest of our outfit, I would be prepared to go anywhere and do anything in the radiographic way with 12 accumulator batteries, and a reliable petrol engine and dynamo that I knew how to work.

No systematic fluorescent screen work and no printing was possible under the conditions that prevailed during the ten months under review, though of course both were occasionally tried in special cases. Eminent surgeons, for one thing. are not prepared to accept the *ipse dixit* of a G. P., and the photographs were permanent records of the evidence available in each instance. It took in the forenoon at least fifteen minutes to sensitise one's retinæ to the fluorescent shadow, and we were liable to interruption any moment to admit patients, the sunlight being as a rule intensely brilliant. The plates used were Ilford X-ray plates, developed with metol and hydroquinone. We found hypono useful till we got a decently plentiful water supply. Most of our early plates were very imperfectly cleaned from hypo, and are now of no value except for domestic purposes. We had a good deal of breakages and many minor accidents. Our plates varied in quality from very thin poor things to many of highest degree of technical excellence, and our average technique has improved from month to month. As the outstanding feature of our work, I regard the habitual and systematic use of a plumb-line to visualise the vertical ray through the radiographic centre of emission. Out of a broom handle and some derelict pieces of wood the orderly improvised a very serviceable apparatus known as the "Heath-Robinson." In whatever way the tube was moved below the table, along or across, the plumb-line moved always in line with the centre of the target—once accurately centred on the screen in the usual way, the upper attachment of the plumb-line was secured by a rubber band. The cord of the plumb-line was made of elastic, so that the sharp, pointed plumb-bob could be brought down on the plates (to make a pinhole in it) or on to the patient for marking him. The occasional occurrence of mysterious marks on our plates remains still unelucidated in some instances. Some of them were probably not X-ray markings, but electrographs. Till January last our work suffered badly from climatic exposure, the wretchedly-fitting glazed sashes of our French carpentered doors and windows having been painted jet black to exclude light. Enough light got in to prevent seeing the image on the screen, but not enough to see to work by. Consequently we had to work *sub Jove* (generally *frigido*) with the sashes open, with ourselves and apparatus exposed to rain, wind, damp, dust and cold, nor was there any efficient means of heating or artificially lighting for many months.

The great difficulty was the risk of *movement* on the part of the patient during the prolonged exposure needed in the use of power supplied from only six accumulators. At least two accumulators were needed to work the interrupter unit; and though the tubes lighted up well at two-fifths of a milliamp, it was difficult to get the wounded patient, *e.g.* in the case of a skull or head, to keep absolutely motionless for the two or three minutes required, nor were we ever free from the risk of the responsive vibration of a shaky floor to the martial tread of an impatient surgeon.

The *quæstio vexata* of X-ray localisation of metallic foreign bodies within the living patient, and the relative advantages of this, that or the other method, must remain an open one till the surgeons have balanced their accounts after the cessation of hostilities. One thing is certain, namely that no one method is universally applicable. There are, in fact, two distinct problems or sets of problems to be resolved. In the first we have a case of recent injury when the parts are bruised, swollen, lacerated, infected, inflamed, and the general condition of the patient pretty bad, and interference is mainly required for conditions in which the presence of the foreign body plays an incidental and comparatively unimportant part. In the second set the parts (it may be after some considerable time) have settled down and become aseptic, the inflammation has subsided, union and cicatrisation have taken place or begun, and the presence of the foreign body remains the chief point of consideration, either in itself or in its interference with function. It will be seen from a note in my table of " plates taken " that the method of exact mathematical localisation was exceptional, nor was I greatly impressed with its relative success as a method. Of the other two methods, the successive exposure of the part on planes at right angles to each other appeared to be the favourite of the responsible surgeons. The stereoscopic method (with its advantage of being able to superpose thc two plates taken and then estimate the relative parallaxes of the foreign bodies and of the identifiable anatomical points) was my own favourite. Unfortunately, only two or three of our surgeons appeared to have the faculty of stereoscopic vision under the conditions of pictorial representation, and to the others it was very difficult to translate my own visual judgments into intelligible anatomical language. Many parts, such as the shoulder, that cannot be taken laterally, lend themselves admirably to stereoscopic methods. In localising mathematically by double exposure on one plate, it was only by chance that the position of the patient was at all the same on the X-ray and on the operating table. In taking a stereoscopic picture I found it of great use to put a distinctively shaped tie-pin on the part between the patient and the tube ; when the pin appeared nearer than the part, one knew at once that one's plates were correctly pared. In localising mathematically by double exposure on one plate, it is of great advantage to use a very small plate, as such can be got quite close to the part, and an angle and part of the adjacent sides can easily be marked on the patient, and not to shift the tube too far. Unfortunately few surgeons would allow the plate to be replaced in contact with the patient on the operation table, nor allow one in every case to put metal rings on the patient's wounds before radiographing, and in fact it was in many cases inadvisable or impossible.

Nor must it be forgotten that in many cases an experienced surgeon will locate his projectile from a single picture 75 times out of a 100, when he has a recent wound track to guide his finger, especially if the foreign body is of any tactile size, and in many cases he is quite content with one picture only.

Without doubt the Röntgen ray is a true *lux in tenebris*. Against the sombre background of the unmerited sufferings of so many of our best and bravest, the present developments of X-ray work (as compared with the infancy of the art) constitute a veritable romance of surgery; one cannot now easily imagine what the surgeons could do without the help afforded them. This is indeed a time of tragic romance; personally two years ago I never dreamed of using upon French soil German rays in the diagnostic aid of British Tommies.

One of the greatest privileges we enjoy out here has been the visiting of similar and superior X-ray installations; every general and many smaller hospitals have their own outfit. There is certainly nothing in Bristol to equal some of these installations. One hears there of much the same and of other difficulties as we had—photographic, electrical and diagnostic—and it was very interesting to learn how different men got over the same difficulties by different devices.

This is not the place nor the time to dilate on the humours of an hospital medical mess on active service. I can only liken it in its candour and cordiality and mutual consideration, when on often harassing duty, with one's old resident hospital days. With heads now grey and bald, and with faces lined and wrinkled, we older members live over again our life of "Auld Lang Syne."

